# A Diffusion Tensor Imaging Study on the White Matter Structures Related to the Phonology in Cantonese–Mandarin Bilinguals

**DOI:** 10.3389/fnhum.2022.851669

**Published:** 2022-05-06

**Authors:** Xiaoyu Xu, Yuying Jin, Ning Pan, Muqing Cao, Jin Jing, Jingwen Ma, Xiaoxuan Fan, Si Tan, Xiaojing Song, Xiuhong Li

**Affiliations:** ^1^Department of Maternal and Child Health, School of Public Health, Sun Yat-sen University, Guangzhou, China; ^2^Guangdong Provincial Maternal and Child Health Care Hospital, Guangzhou, China; ^3^Wuhan Children’s Hospital, Tongji Medical College, Huazhong University of Science & Technology, Wuhan, China

**Keywords:** Cantonese–Mandarin bilinguals, diffusion tensor imaging, phonological processing, tractography, tract-based spatial statistic

## Abstract

Cantonese and Mandarin are logographic languages, and the phonology is the main difference between the two languages. It is unclear whether the long-term experience of Cantonese–Mandarin bilingualism will shape different brain white matter structures related to phonological processing. A total of 30 Cantonese–Mandarin bilinguals and 30 Mandarin monolinguals completed diffusion-weighted imaging scan and phonological processing tasks. The tractography and tract-based spatial statistics were used to investigate the structural differences in the bilateral superior longitudinal fasciculus (SLF), inferior longitudinal fasciculus (ILF), and inferior fronto–occipital fasciculus (IFOF) between Cantonese–Mandarin bilinguals and Mandarin monolinguals. The *post-hoc* correlation analysis was conducted to investigate the relationship between the different structures with phonological processing skills. Compared to the Mandarin monolinguals, the Cantonese–Mandarin bilinguals had higher fractional anisotropy (FA) along the left ILFs higher mean diffusivity (MD) along the right IFOF and the temporoparietal segment of SLF (tSLF), higher axial diffusivity (AD) in the right IFOF and left ILF, and lower number of streamlines in the bilateral tSLF. The mean AD of the different voxels in the right IFOF and the mean FA of the different voxels in the left ILF were positively correlated with the inverse efficiency score (IES) of the Cantonese auditory and Mandarin visual rhyming judgment tasks, respectively, within the bilingual group. The correlation between FA and IES was different among the groups. The long-term experience of Cantonese–Mandarin bilinguals shapes the different brain white matter structures in tSLF, IFOF, and ILF. Compared to the monolinguals, the bilinguals’ white matter showed higher diffusivity, especially in the axonal direction. These changes were related to bilinguals’ phonological processing.

## Introduction

Globally, there are 55 countries with more than one official languages, and immeasurable regions with only one official language but also regional and local languages^1^. Understandably, countless people use more than one languages frequently for a long time. The long-term bilingual experience affects multiple cognitive functions (see a review Costa and Sebastian-Galles, 2014). The effect of bilingualism on the phonological processing received much attention. Bilinguals rely on one brain to process two distinct phonologies. The functional imaging studies showed that the brain activation patterns during phonological processing of the native and the second language (L1 and L2, respectively) for bilinguals were different (see reviews Liu and Cao, 2016; Sulpizio et al., 2020), as well as the brain activation pattern during phonological processing of either language for bilinguals was different from that of the single language for monolingual (Parker Jones et al., 2012; Cao et al., 2013; Ma et al., 2020). The functional changes are accompanied by anatomical changes (see reviews Li et al., 2014; Stein et al., 2014). The white matter coordinates communications between the different brain regions (Douglas, 2008) and interacts with the cortex function (Duffau, 2015). The white matter has lifelong plasticity (Gibson et al., 2014; McKenzie et al., 2014; Fields, 2015) and is likely to be modulated by environmental stimulus and behavioral experience, such as bilingualism (Li et al., 2014). Therefore, it is reasonable to hypothesize that the long-term bilingual experience can shape different white matter structures of phonological-related tracts.

The diffusion tensor imaging (DTI) technique allowed a detailed observation of white matter structure *in vivo* without invasion (Basser and ÖZarslan, 2014). The characteristics of white matter can be described by the DTI indices, such as fractional anisotropy (FA) and diffusivity measurements including mean, axial, and radial diffusivities (MD, AD, and RD, respectively) (Soares et al., 2013). According to the dual-stream model of the language processing, the dorsal cortical circuit including the perisylvian language areas is demonstrated to be involved in processing the phonological information in written and spoken languages (Hickok and Poeppel, 2007; Schlaggar and McCandliss, 2007). The fiber dissection studies claimed that the superior longitudinal fasciculus (SLF) is the core fiber connecting the dorsal language cortical regions (Sarubbo et al., 2016). On the other hand, the ventral cortical circuit is generally considered to play an important role in semantic processing. The core tracts connecting the ventral stream consist of the ILF and the inferior fronto–occipital fasciculus (IFOF) (Sarubbo et al., 2016). However, there is also some evidences supporting that the ventral stream has a relationship with phonology. Li et al. (2017) found patients with lesion in the left IFOF had worse performance in the phonological fluency task. The DTI studies also reported that the structure of right ILF/IFOF was related to pseudo word reading and decoding (Lebel et al., 2013), and the structure of left IFOF was related to rapid automatized naming and non-word reading (Rollans et al., 2017). The researchers claimed that the two tracts might play a role in the mapping from orthography to phonology (Rollans et al., 2017).

Accumulating DTI evidence revealed that the alphabetic–alphabetic bilinguals had different white matter structures from their monolingual peers, especially in the SLF, ILF, and IFOF (Luk et al., 2011; Schlegel et al., 2012; Gold et al., 2013; Pliatsikas et al., 2015; Kuhl et al., 2016; Singh et al., 2017; Anderson et al., 2018a). However, the reproducibility of these studies is low. It is considerable because bilingualism is a multifaceted construct sensitive to age of L2 acquisition (AoA), L2 immersion time, and language categories, etc. (Li et al., 2014). Most of the current DTI studies on bilingualism recruited the first-generation immigrants or local L2 learners as the bilingual samples, who generally learn L2 lately and use L2 for a short time (Schlegel et al., 2012; Cummine and Boliek, 2013; Pliatsikas et al., 2015; Kuhl et al., 2016; Rossi et al., 2017). In the SLF, short-term late bilinguals usually demonstrated higher FA and lower RD than monolinguals (Schlegel et al., 2012; Pliatsikas et al., 2015; Rossi et al., 2017). This is similar to the white matter characteristics of the subjects after L2 training (Hosoda et al., 2013; Mamiya et al., 2018). However, for bilinguals born in bilingual societies, they learn L2 at an early age, and the stimulus of the two languages is long-term and continuous. Only a few DTI studies on bilingualism recruited long-term bilinguals who learned L2 at an early age (Luk et al., 2011; Singh et al., 2017; Anderson et al., 2018a). Singh et al. (2017) and Anderson et al. (2018a), respectively, recruited lifelong bilingual samples from Canada and Hindi, and matched them well with the monolingual samples. Both reported that the bilinguals had higher AD in the SLF than the monolinguals, and Singh et al. also reported that the bilinguals had higher MD in the SLF than the monolinguals. This is inconsistent with the results of the studies focusing on late short-term bilinguals (Schlegel et al., 2012; Pliatsikas et al., 2015; Rossi et al., 2017). It is speculated that the long-term immersion in a bilingual environment from an early age might induce different effects from relatively short-term L2 experience (Singh et al., 2017). Globally, there are countless regions with more than one language (see footnote 1), and nearly 66% of people are raised as bilingual speakers from childhood and use more than one language for a long time (Marian and Shook, 2012). In this study, the white matter structural characteristics of such long-term bilinguals are of our interest.

In addition, some rare studies focused on the logographic bilinguals. The literature (Li et al., 2014) indicated that the language typology could affect the changes in bilinguals’ brain structure. Different from alphabetic languages, Chinese is a unique language best known for its logographic writing system (Deng et al., 2013). The orthography-to-phonology mapping rule is extremely opaque in Chinese, while it is relatively transparent in alphabetic languages. Thus, the Chinese readers tend to adopting the addressed phonology strategies, which means directly retrieving stored phonological representations for the whole word (Cao et al., 2017). In monolinguals, the evidence from fMRI studies suggested that compared to alphabetic languages, reading Chinese requires extra involvement of cortex in the ventral stream [for details see the references (Bolger et al., 2005; Tan et al., 2005; Sun et al., 2011)]. For bilinguals, when processed as the L2 in the phonological tasks, compared to alphabetic language, Chinese processing also need greater activation in the ventral cortical regions including fusiform gyrus, inferior temporal lobe and occipital regions (Kim et al., 2016; Cao et al., 2017). As claimed, the ventral cortical regions play a role in mapping orthography to addressed phonology, which is essential in reading Chinese. The ILF and IFOF are the two core tracts connecting the ventral cortex (Sarubbo et al., 2016). Qi et al. (2015) found that FA and RD of the right ILF could predict the Chinese achievement of native English speakers after short-term Chinese training. Besides, Cummine and Boliek (2013) observed Chinese–English bilinguals had different DTI indices in the bilateral IFOF and right ILF from English monolinguals. These two studies suggested that using Chinese and another alphabetic language together might be related to the white matter tracts in the ventral pathway. However, there has been no study focusing on the whiter matter characteristics of bilinguals using two kinds of logographic languages. In addition, none of these two studies focused on long-term bilinguals who learn L2 at an early age. Thus, we aim to investigate how the long-term bilingual experience of two logographic languages may shape the white matter structure. We hypothesized that the white matter structure in the SLF, ILF, and IFOF might exhibit an effect.

Mandarin and Cantonese are the two major Chinese and both are logographic languages. In Guangdong Province, China, nearly half of the population speaks both Cantonese and Mandarin (Huiming and Zhe, 2016). For Cantonese–Mandarin bilinguals, Cantonese is their native language (L1) mainly for daily communication and local medium, while Mandarin is the L2 for formal situations. As the official language, Mandarin is popularized nationwide throughout China. Mandarin is the teaching language in Guangdong, and the children learn how to pronounce and write in Mandarin from primary school or even earlier. In terms of the linguistic characteristics, Cantonese and Mandarin share the same set of characters and have similar grammatical structures (Tardif et al., 2009). However, they shared the same pronunciation for only 21.5% of characters (Li, 1990). Thus, although Cantonese is defined as a dialect according to socioeconomic factors, it is still deemed as an individual language in the region of psycholinguistics (Chen et al., 2004; Tardif et al., 2009). The evidence from behavioral studies suggested that Cantonese–Mandarin bilinguals performed different phonological processing skills compared to their Mandarin monolingual peers over a wide age span (Chen et al., 2004; Li et al., 2011). Our studies on functional magnetic resonance imaging (fMRI) showed that Cantonese–Mandarin bilinguals had different brain activation patterns and functional connectivity related to phonological tasks from Mandarin monolinguals, such as the inferior frontal gyrus and angular gyrus (Ma et al., 2020, 2022). We also observed different functional connectivity in phonology-related subnetwork between Cantonese–Mandarin bilinguals and Mandarin monolinguals through resting-state MRI, such as the resting-state functional connectivity of the inferior frontal gyrus and angular gyrus with temporal regions (Fan et al., 2021). Therefore, it is assumed that the white matter structure related to the phonology might be different between Cantonese-Mandarin bilinguals and Mandarin monolinguals, and the different structure might have a relationship with phonological processing.

In this study, we used the tractography and tract-based spatial statistics (TBSS) analysis to test our hypothesis. A binary mask of tracts of interests (TOI) was constructed for TBSS analysis to improve the anatomical accuracy and the power to detect significance (Hamalainen et al., 2017). We measured the phonological processing skills and performed a *post-hoc* correlation between the phonological processing skills and the significant regions revealed in the group-wise comparison. The *post-hoc* comparison could help us better understand the white matter plasticity induced by Cantonese–Mandarin phonological processing. The aim of this study was listed as follows: (1) To test whether the white matter structure in SLF, ILF, and IFOF is different between Cantonese–Mandarin bilinguals and Mandarin monolinguals; (2) To explore the relationship between the phonological processing skills and the significant regions in the white matter structure.

## Materials and Methods

### Participants

A total of 31 Cantonese–Mandarin bilinguals and 30 native Mandarin monolinguals were recruited from Canton. All the subjects underwent diffusion-weighted imaging (DWI) and T1-weighted scans. The bilinguals in this study were born in the Guangdong province of mainland China, a Cantonese–Mandarin bilingual society. Cantonese was their native language, and they learned Mandarin at the preschool or elementary school stage. For 27 of the 30 Mandarin monolinguals were born and grew up in Mandarin regions with Mandarin as the native language. Three of the monolinguals grew up in families of Shandong, Henan or Sichuan dialects which were the variants of Mandarin and can be fully interconnected with Mandarin (Institute of Language et al., 2012). None of the monolinguals had learned or used Cantonese or any Chinese southern dialect. One participant in the bilingual group was excluded because his DWI image was incomplete. Finally, we included 30 Cantonese–Mandarin bilinguals [6 males; age (*y*), *mean* ± *standard* deviation (*M* ± *SD*), 21.17 ± 1.97)] and 30 Mandarin monolinguals [9 males; age (*y*), *M* ± *SD*, 21.40 ± 2.03] in analysis. The bilinguals’ second language (L2) acquisition age ranged from 3 to 7 years old. The proficiency of the languages of participants was evaluated referring to the language and social background questionnaire (LBSQ). The LBSQ is a self-assessment tool and has been proven to be reliable and valid in diverse languages (Anderson et al., 2018b). Also, since English is a compulsory course in the Chinese education system, all participants in this study had English learning experiences. We used the grades of College English Test Band 4 (CET4), a national English test in China for undergraduate and postgraduate students, to evaluate participants’ English proficiency and included CET4 grades as a nuisance variable in the group-wise comparison. The participants’ non-verbal intelligence quotient evaluated by Raven’s Standard Progressive Matrices Test was matched in the two groups.

All participants were right-handed referring to the Edinburgh Handedness Inventory (Oldfield, 1971). The ones with learning disabilities, neurological diseases, psychiatric disorders, visual and hearing difficulties, attention deficit hyperactivity disorder, or contraindications of MRI were excluded. All participants signed written informed consent before participating. The Medical Ethics Committee, Sun Yat-sen University provided ethical approval for this study with the ethical approval number [L2016] No. 036.

### Behavioral Measures of Phonological Processing Skills

We chose the rhyming judgment task, the rapid automatized naming (RAN) task, and the digit span test to assess the three aspects of phonological processing skills (Wagner and Torgesen, 1987), respectively. For bilinguals, we performed the tasks in both Mandarin and Cantonese, while only in Mandarin for monolinguals.

#### Rhyming Judgment Task

The visual and auditory rhyming tasks were performed to test the phonological awareness of getting sound-based representations from the written and spoken words. In the visual task, the participants needed to transfer the orthography to phonology, while they did not need it in the auditory task. Both the rhyming tasks were displayed on Eprime2.0 based on previous studies on Chinese adults (Cao et al., 2017). Each task consisted of 30 trials. The design of the tasks was displayed in Supplementary Figure 1. All the words used in the tasks consisted of one onset and one rhyme and the two paired words had the same tone. The participants were asked to judge whether the two paired words had the same rhyme. The characters used in the visual rhyming judgment tasks were the common words chosen from the Modern Chinese Dictionary and the Cantonese Dictionary, respectively. The average reaction time (RT) of responses and the accuracy rate (AR) were recorded for either task. An integrated variable called inverse efficiency score (IES) was computed by dividing RT by AR (IES = RT/AR) (Bruyer and Brysbaert, 2011) and used as the measurement of rhyming judgment task performance.

#### Rapid Automatized Naming

The classical RAN was used to assess the ability of phonological lexical retrieval (Siddaiah et al., 2016). We used digits and objects as materials. 40 symbols were printed on A4-sized paper and participants were asked to read them twice as fast and accurately as possible. The average RT (ms) of RAN was calculated by dividing the total RT (ms) by the mean number of correct reactions.

#### Digit Span Test

The Digit Span test from the Wechsler Adult Intelligence Scale-Revised (WAIS-R) Chinese version was used to assess verbal working memory ability. The number of correct answers was recorded as the total score.

### Magnetic Resonance Imaging Acquisition and Analysis

#### Magnetic Resonance Imaging Acquisition

Diffusion-weighted images (DWI) were acquired by a 3.0 T Siemens Scanner (Siemens Healthcare, Erlangen. Germany) at the Hunan Normal University in Guangzhou. A single-shot spin–echo echoplanar imaging sequence was used with the following parameters: Repetition time (TR) 10,000 ms; echo time (TE), 90 ms; flip angle,90°; matrix size = 128 × 128; field of view, 256 mm; voxel size 2 mm × 2 mm × 2 mm; *b* = 1,000 s/mm^2^; number of average, 1; and GRAPPA factor, 2. The diffusion gradients in 64 non-collinear directions with one image of *b* = 0 s/mm^2^ were collected.

The T1-weighted 3D images were acquired using magnetization prepared rapid gradient–echo sequence with following parameters: TR = 1,900 ms; TE = 2.52 ms; flip angle = 90°; matrix size = 256 × 256; field of view = 256 mm; voxel size 1 mm × 1 mm × 1 mm; number of average, 1.

#### An Magnetic Resonance Imaging Preprocessing and the Tractography

An MRI preprocessing was performed using FSL (version 5.0.9) (FMRIB Software Library^2^). The procedures included visual inspection of quality control, eddy correction (Andersson and Sotiropoulos, 2016), deletion of non-brain tissue (Smith, 2002), calculation of DTI to get FA, MD, AD, and RD maps (Behrens et al., 2003). Additionally, DTI data quality was quantificationally evaluated according to the log file generated in eddy correction using trac-all qa tool (Yendiki et al., 2014) in FreeSurfer. The mean of average translation (mm) was 0.76 ± 0.24 for bilinguals and 0.78 ± 0.21 for monolinguals, while the mean of average rotation (×10^–1 °^) was 0.47 ± 0.12 for bilinguals and 0.46 ± 0.10 for monolinguals. The percentage of bad slices for both groups was 0 and the average signal drop-out score was 1. No difference was found in DWI quality measures between the two groups.

Tractography was performed using diffusion toolkit and trackvis^3^, with the following parameters: Interpolated streamline method; minimum FA threshold, 0.2; step length, 0.5 mm; maximum angle threshold,35°. To minimize the subjective variation of manual regions of interest (ROI) definition, we used the semiautomatic method to define ROIs according to Su et al. (2018). We followed the acknowledged protocol to dissect ILF and IFOF (Catani and Thiebaut de Schotten, 2008). As there were multiple names of the SLF subdivisions, which could confuse the readers, we followed the classification proposed by Nakajima et al. (2019) to divide the SLF to the following four parts: Dorsal SLF, ventral SLF, AF, and temporoparietal segment of SLF (tSLF). Since the dorsal and ventral SLF are difficult to be divided using DTI model (De Schotten et al., 2011), and the functions of them are highly correlated (Budisavljevic et al., 2017; Nakajima et al., 2019), we reconstructed them together following the protocol proposed by De Schotten et al. (2011) and used frontoparietal segment of SLF (fSLF) to referred the merged tract. The AF and tSLF were reconstructed following the protocols proposed by Catani et al. (2005). The mean FA, MD, AD, and RD of each tracts were extracted. We also recorded the number of streamlines per tract as well as of the whole brain. No difference was found in the number of streamlines in the whole brain between groups (*M* ± *SD*; bilinguals, 91316.8 ± 26705.1; monolinguals, 87079.0 ± 8899.8, *p* = 0.416).

#### Magnetic Resonance Imaging Data Processing

We performed tract-specific TBSS analysis to compare white matter structures of the TOI between the two groups. Every subject’s binary mask of TOI was generated using the fslmaths tool in native space and registered to the MNI space. Then, we create an averaged binary mask of TOI by including the voxels belonged to 75% participants. The TBSS analysis was performed according to the standard pipeline supplied in FSL website (Smith et al., 2006). The averaged binary mask was used in the permutation tests. Threshold-free cluster enhancement (Smith and Nichols, 2009) with 10,000 permutations was set. The family-wise error rate (FWR) was corrected. The location of significant voxels in the tract-specific analysis was reported through the averaged binary masks of separate tracts of TOI. The mean values of the different DWI parameters of each significant cluster was extracted using fslmeants tool for the *post hoc* association analysis.

We also performed a whole-brain-level TBSS analysis to explore the group-wise difference outside the TOI and facilitate the comparison with the previous studies. As this is beyond our scope of work, the results were provided in the Supplementary Materials.

### Statistical Analysis

Statistical analysis was performed using R4.0.2. The comparison of demographic information and behavioral measures between the bilingual group and monolingual group was conducted by the independent-samples *t*-tests, Mann–Whitney *U* tests, or Chi-squared test. Wilcoxon paired-samples signed rank tests were performed for Cantonese and Mandarin proficiency and phonological processing skills comparison within the bilingual group. The uncorrected *p*-values were reported and compared with Bonferroni-corrected α.

We used linear mixed models to test the bilingualism effect on the white matter structure of TOI. The linear mixed models allow random effects induced by inter-subject variability to be incorporated into the model. The DTI metrics (FA, MD, AD, and RD) as well number of streamlines were set to dependent variables. Group, tract as well as group by tract interaction were defined as the fixed effects. Subjects and English proficiency were defined as random effects. The R package of lmerTest (Kuznetsova et al., 2017) was used and the formula was as follows: Structural indices ∼ Group × Tract + (1| Subjects) + (1| English). When the significant Group × Tract interaction was observed, we conducted a *post hoc* analysis of covariance (ANCOVA) to compare the structural indices between groups. English proficiency was defined as the covariate. The concerning the family-wise error (FWE), a partial Bonferroni correction was performed according to the procedure published on the SISA website^4^. The corrected α were 0.018, 0.020, 0.009, 0.019, 0.007, respectively, for FA, MD, AD, RD, and the number of streamlines.

For *post-hoc* subgroup correlation analysis, Pearson correlation analysis, and Spearman rank correlation analysis were performed separately for variables in normality and non-normality. The variables for Spearman rank correlation were RT of the Mandarin RAN digit task, IES of the Cantonese auditory rhyming task, IES of the Cantonese visual rhyming task within the bilingual group, and none in the monolingual group. The partial Bonferroni method was used to perform multiple comparison corrections. The corrected α was 0.010 for the bilingual group, and 0.014 for the monolingual group. The interaction effect was tested, if there was a significant correlation between structural indices and Mandarin behavioral performance. The correlation coefficients with an uncorrected *p* were presented in the Supplementary Table 1, but those correlations survived the FWE correction were depicted in Figure 3.

**FIGURE 1:**
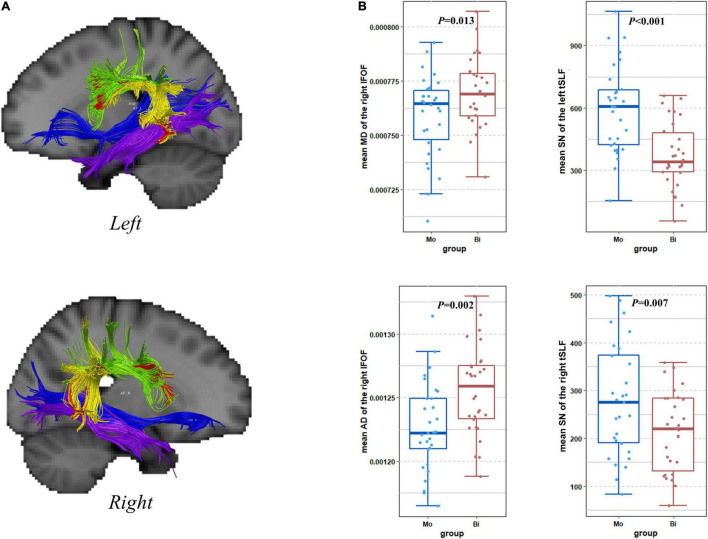
Results of tractography. **(A)** The five TOIs: fSLF (green), AF (red), tSLF (yellow), ILF (purple), and IFOF (blue) overlaid on a standard MNI brain. **(B)** The boxplots showing group-wise differences in mean MD, AD, and number of streamlines from tracts. Mo, Monolingual group; Bi, Bilingual group; SN, number of streamlines.

**FIGURE 2:**
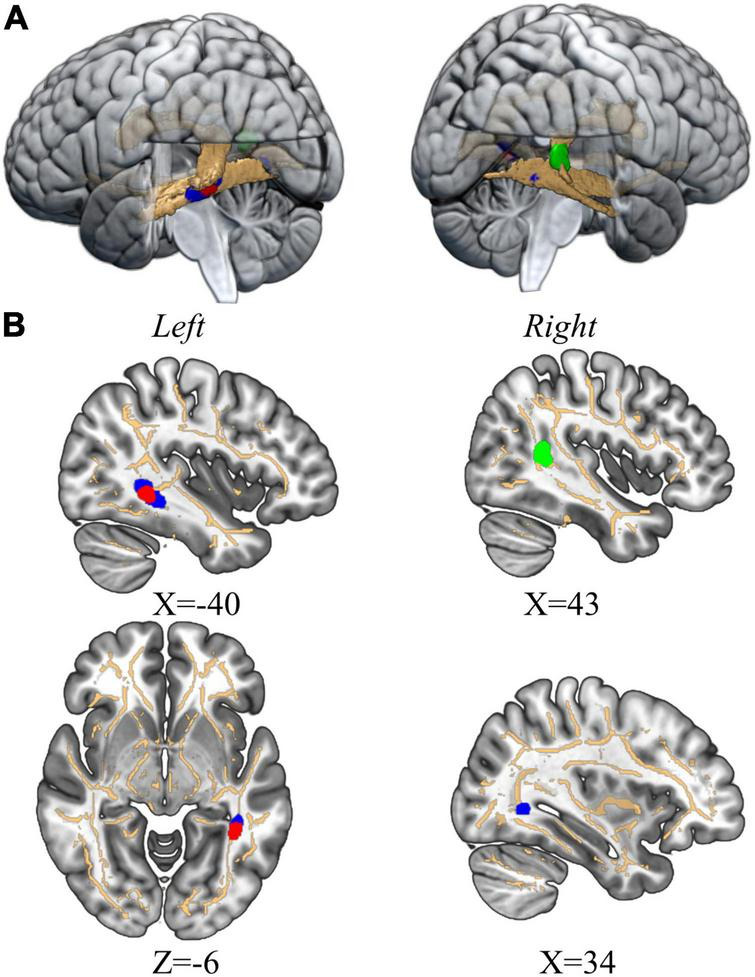
The significant clusters in the tract-specific TBSS analysis. **(A)** The 3D view. The binary mask of TOI is copper while the significant clusters in the comparison of FA, MD, and AD is in red (FA), green (MD), and blue (AD) respectively. **(B)** The cross-section view. The mean FA skeleton is copper and significant clusters emphasized *via* TBSS-fill is set as the same color in 3D view.

**FIGURE 3:**
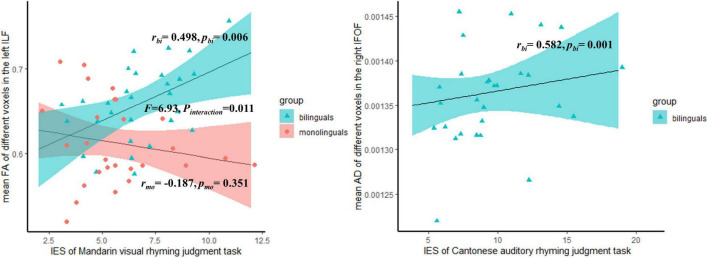
The significant correlation between the mean different DTI indices of significant voxels in the tracts and behavioral measures, within the subgroups.

## Results

### Phonological Processing Skills

The performance of phonological processing skills was reported in Table 1. The CC (Cantonese–Mandarin bilinguals who were performing the tasks in Cantonese) demonstrated a higher IES than the CM (Cantonese–Mandarin bilinguals who were performing the tasks in Mandarin), and the MM (Mandarin monolinguals performing the tasks in Mandarin) in both visual and auditory rhyming judgment tasks (*p* < 0.017). The CM also showed higher IES than the MM in the visual rhyming judgment task (CM: 6.74 ± 1.93 *vs.* MM: 5.69 ± 2.29, *p* = 0.017). The higher the IES, the poorer the phonological awareness. Furthermore, the CC got a higher score in the digit span task than the CM (CC: 33.17 ± 3.37 *vs.* CM: 33.17 ± 3.37, *p* = 0.005). The CM performed slower than the CC and the MM, in the digit RAN task (CM: 0.30 ± 0.06 *vs.* CC: 0.28 ± 0.04, and MM: 0.26 ± 0.04, *p*_CM–CC_ = 0.001, *p*_CM–MM_ = 0.015).

**TABLE 1:** Demographic characteristics and phonological processing skills.

	Bilinguals (*N* = 30)		Monolinguals (*N* = 30)					
	Mandarin	Cantonese	Mandarin		*p*	*p*_1_	*p*_2_	*P*_3_
Age (*M* ± *SD*, years)	21.17 ± 1.97		21.40 ± 2.03		0.653	–	–	–
Gender (female/male, *N*)	24/6		21/9		0.552	–	–	–
IQ score (*M* ± *SD*)	122.40 ± 11.86		124.33 ± 14.16		0.569	–	–	–
Acquisition age (*M* ± *SD*)	4.53 ± 1.33	1.15 ± 0.83	1.73 ± 1.13		–	**<0.001**	**<0.001**	0.067
***Socio–economic status (N)***								
Education (undergraduate/postgraduate)	22/8			23/7	0.766	–	–	–
District of residence (city/suburb/missing)	19/10/1			20/10/0	0.926	–	–	–
Father’s education					0.894	–	–	–
Junior high school or below	13			11				
Senior high school or technical secondary school	11			12				
College or above	6			7				
Mother’s education					0.364	–	–	–
Junior high school or below	11			13				
Senior high school or technical secondary school	16			11				
College or above	3			6				
***Language proficiency (M ± SD)***								
Speaking^2^	8.50 ± 1.11	9.43 ± 0.77		8.87 ± 1.20		0.162	**0.001**	0.053
Writing^2,3^	8.57 ± 1.14	5.73 ± 2.13		9.03 ± 0.93		0.433	**<0.001**	**<0.001**
Reading^2,3^	8.87 ± 0.97	7.77 ± 1.43		8.77 ± 1.17		0.479	**<0.001**	**0.003**
Comprehension	8.83 ± 1.12	8.83 ± 0.91		8.77 ± 1.28		0.785	0.941	0.926
CET4 grades*	557.00 ± 45.98			517.78 ± 60.69	**0.007**	–	–	–
***Phonological processing skills [M ± (SD)]***								
Visual rhyming task IES^2,3^	6.74 ± 1.93	8.34 ± 3.15		5.69 ± 2.29		**0.017**	**<0.001**	**0.008**
Auditory rhyming task IES^2,3^	8.36 ± 3.45	10.14 ± 4.25		7.17 ± 2.18		0.270	**<0.001**	**0.003**
Digits span score^2^	31.27 ± 3.87	33.17 ± 3.37		32.27 ± 4.43		0.277	**0.005**	0.380
RAN-digits RT (ms)^1,2^	0.30 ± 0.06	0.28 ± 0.04		0.26 ± 0.04		**0.001**	**0.015**	0.019
RAN-object RT (ms)	0.58 ± 0.09	0.59 ± 0.11		0.57 ± 0.08		0.906	0.473	0.548

p_1_ is the significance of the comparison between Cantonese–Mandarin bilinguals performing the tasks in Mandarin (CM) and Mandarin monolinguals performing the tasks in Mandarin (MM). p_2_ is the significance of the comparison between CM and Cantonese–Mandarin bilinguals performing the tasks in Cantonese (CC). p_3_ is the significance of the comparison between the CC and MM. α was 0.05 for variables in only two groups and was corrected by Bonferroni’s correction for the comparisons of variables in all three groups (α_corrected_ = 0.05/3 = 0.017). Superscript 1, 2, 3 separately mean the p1, p2, p3 were under the significant threshold. Asterisk (*) means that the “r” value is significant.

### The Results of Tractography

The tractography of fSLF, tSLF, ILF, and IFOF succeeded in all the subjects, but AF known not to be traceable in all individuals (Catani et al., 2007; Vanderauwera et al., 2017) was failed to be reconstructed in a few subjects. The right AF could not be reconstructed in 13 bilinguals and 3 monolinguals, while the left AF could not be reconstructed in 5 bilinguals and 2 monolinguals. The tractography reconstructions were shown in Figure 1A.

We used the linear mixed models to test the effect of the group on the white matter structure of TOI. The DTI metrics including FA, MD, AD, and RD, as well as the number of streamlines were treated as the dependent variable. The group, tract (5 TOI × 2 hemispheres) and the interaction term were set to fixed factors. In the FA model, neither significant group effect nor group × tracts interaction was found. In the MD model, the group effect was non-significant, but the group × tracts interaction was significant (*T* = 2.31, *p* = 0.021). The *post hoc* ACNOVA revealed a difference in the right IFOF [*M ± SD* (×10^–4^), bilinguals: 7.77 ± 0.32, monolinguals: 7.59 ± 0.19, *p* = 0.013] after controlling English proficiency. In the AD model, the interaction was non-significant, but the group effect was close to the significant threshold (*T* = 1.85, *p* = 0.065). We also performed the *post-hoc* ACNOVA to investigate the group-wise difference in each tract. Only in the right IFOF was the difference found after multiple comparison correction [*M* ± *SD* (×10^–4^), bilinguals: 12.56 ± 0.34, monolinguals: 12.27 ± 0.35, *p* = 0.002]. In the RD model, the group effect was non-significant, but the interaction was significant (*T* = 2.07, *p* = 0.039). However, the *post hoc* tests revealed no significant difference in each tract. In the number of streamlines model, there was a significant interaction effect (*T* = –2.11, *p* = 0.036) with no group effect. The *post hoc* ACNOVA revealed group-wise differences in the bilateral tSLF (*M* ± *SD*, left tSLF: bilinguals: 371.83 ± 158.32, monolinguals: 592.57 ± 0.34, *p* < 0.001; right tSLF: bilinguals: 212.13 ± 82.85, monolinguals: 292.18 ± 137.79, *p* = 0.007). The box plots of the significant differences in TOI were presented in Figure 1B.

### The Results of Tract-Specific Tract-Based Spatial Statistic Analysis

The tract-specific TBSS analysis revealed that compared to the Mandarin monolinguals, Cantonese–Mandarin bilinguals had higher MD in the right tSLF, higher FA in the left ILF and higher AD in the left ILF and right IFOF after controlling the English proficiency and average translation (*p* < 0.05, FWE corrected). The results are represented in Figure 2, and the peak coordinates and number of the differential voxels distributed in each tract are reported in Table 2. Monolinguals did not show higher FA, MD or AD than bilinguals in any voxel. In addition, RD did not yield significance in the tract-specific TBSS analysis.

**TABLE 2:** Results of the tract-specific TBSS analysis.

White matter tract	DTI parameter	Voxels	Peak coordinate	*p*_min_
tSLF (R)	MD (Bilingual > Monolingual)	39	(43, –48, 8)	0.011
ILF (L)	FA (Bilingual > Monolingual)	24	(–40, –45, –5)	0.018
ILF (L)	AD (Bilingual > Monolingual)	75	(–41, –43, –7)	0.005
IFOF(R)	AD (Bilingual > Monolingual)	10	(34, –57, 0)	0.032

*CET4 grades and average translation (mm) were controlled, with FWE corrected and a lowest threshold of 10 voxels for each cluster. tSLF, temporoparietal segment of superior longitudinal fasciculus; ILF, inferior longitudinal fasciculus; IFOF, inferior fronto–occipital fasciculus; L, left; R, right.*

### The Results of *post- hoc* Correlation Analysis

Only those significant clusters identified in the TBSS analysis were found to correlate with the behavioral performance. As shown in Figure 3, the mean FA of the different voxels in the left ILF was positively correlated with Mandarin visual rhyming judgment task IES only within the bilingual group (*r* = 0.498, *p* = 0.006), but it does not correlate within the monolingual group (*F* = 6.93, *p*_interaction_ = 0.011). The mean AD of the different voxels in the right IFOF was positively correlated with the IES of Cantonese auditory rhyming judgment task within the bilingual group (*r* = 0.582, *p* = 0.001). The detailed correlation coefficients and *p*-values of all the behavioral measures were provided in Supplementary Table 1.

## Discussion

We combined the tractography and TBSS to investigate the difference in white matter structures along the bilateral SLF, ILF, and IFOF between Cantonese–Mandarin bilinguals and Mandarin monolinguals. We observed that compared to monolinguals, bilinguals had higher MD along the right tSLF and IFOF, higher AD along the left ILF and right IFOF, higher FA along the left ILF, as well as fewer streamlines in the bilateral tSLF. Furthermore, there was a significant relationship between the phonological awareness and the mean DTI indices of the different voxels along the ventral tracts. Besides, the correlation between the DTI indices and the behavioral performance was different between the two groups. The results support our hypothesis. The current study was the first study to explore the difference in the white matter structures related to phonology between Cantonese-Mandarin bilinguals and Mandarin monolinguals.

### Differences in Phonological Processing Skills

First, we observed that Cantonese–Mandarin bilinguals had worse Cantonese phonological awareness than Mandarin, in both visual and auditory rhyming tasks. It is reasonable, considering that Cantonese–Mandarin bilinguals in mainland China only accept *pinyin* instruction for Mandarin but does not accept Cantonese in school. *Pinyin* is the phonological coding system used in China. As both Mandarin and Cantonese are morphosyllabic, which are not divisible at the phoneme, *pinyin* instruction can greatly facilitate the phonological awareness among the Chinese (Shu et al., 2008). The deficiency of *pinyin* instruction for Cantonese may lead to the poorer Cantonese phonological awareness of the bilinguals. In addition, Cantonese–Mandarin bilinguals had better digit span and RAN performance in Cantonese than Mandarin. This phenomenon is consistent with previous evidence that bilinguals have better abilities of working memory (Grundy and Timmer, 2016) and phonological lexical retrieval (Pennino, 2010; Yeung, 2016) in their native languages than their L2.

Second, we observed that the Cantonese–Mandarin bilinguals performed worse in the Mandarin visual rhyming judgment task and digit RAN task than Mandarin monolinguals. The lower speed of naming is probably related to the interference from non-target language when bilinguals are facing language choosing (Jian et al., 2011). The differences will be discussed combined with the neuroimaging results below.

### Dorsal White Matter Tracts

First, we found that Cantonese–Mandarin bilinguals had higher MD in the right tSLF and lower number of streamlines in the bilateral tSLF than Mandarin monolinguals. The MD measures the average diffusivity of water molecules across all directions, namely isotropy, and higher MD reflects more free water diffusion in white matter (Soares et al., 2013). The literature showed that the higher MD can accompany increased axonal caliber, more fiber crossings, looser packing density, fewer synapses or glial cells (Sagi et al., 2012), or even an increase in tissue water content, such as increased cerebral blood flow (Jin and Kim, 2008). The fewer streamlines are usually interpreted as lower fiber counts corresponding to lower connectivity strength between two brain regions (Tsang et al., 2017; Young et al., 2018). However, the DTI metrics and number of streamlines are also affected by many confounding effects such as curvature, length, branching, and fiber crossings (Jones et al., 2013). The interpretation from DTI metrics to specific anatomical microstructures should be treated prudently. One possibility accounting for the higher MD and lower number of streamlines is that the bilinguals had looser packing density and lower connectivity strength in the tSLF. Another possibility is that bilinguals had higher fiber complexity originating from fiber branching and crossings.

Different from our finding, some intervention studies observed increased FA in the right SLF for L2 learners (Hosoda et al., 2013; Mamiya et al., 2016). For example, Hosoda et al. (2013) found that FA in the right SLF would increase after 16 weeks of English training for Japanese speakers. Mamiya et al. (2016) also found that FA in the right SLF was positively correlated with the number of training days in English immersion program for Chinese speakers. Animal models showed that the increase of FA was associated with learning (Gibson et al., 2014). Hosoda and Mamiya guessed that the increase of FA might be associated with increased brain myelination. However, we believed that our results are less likely to reflect remodeling of myelin, because myelination is not a necessary condition for MD changes (Takeuchi et al., 2015) or number change of streamlines (Jones et al., 2013). Furthermore, we found that there was no significant difference in FA and RD in the right SLF between the two groups. The FA and RD are sensitive to myelin changes (Song et al., 2002; Basser and Pierpaoli, 2011). The L2 training in both the intervention studies was short-term, whereas the Cantonese–Mandarin bilingual adults in our study use L2 regularly since an average age of 4.53 (in the range of 3–7 years). The evidence from motor training suggested that short-term intervention increased FA (Scholz et al., 2009), while compared with short-term intervention, long-term training would induce increased diffusivity, and reduced fiber coherence (Giacosa et al., 2019). Accordingly, we speculated that different from short-term training, lifelong bilingual language experience would generate greater isotropic diffusivity. Consistent with our finding, Singh et al. (2017) reported that Hindi–English bilinguals had higher MD in the bilateral SLF and higher AD in the right SLF than Italian monolinguals. The Hindi–English bilinguals also lived in bilingual society and started to use L2 frequently since an average age of 5 years. They suggested that lifelong bilingual language experience would generate greater isotropic diffusivity.

Anderson et al. (2018a) observed higher AD in the left SLF for lifelong bilinguals in Canada compared to monolinguals after carefully matching between groups in age and general cognitive abilities. However, they did not compare the MD values between the groups. The MD is the average of the dispersion of water molecules in each direction, including the radial and axial (Soares et al., 2013). The AD is highly correlated to MD. Thus, it is somewhat possible that the bilinguals and the monolinguals in Anderson’s study also had a difference of MD in the left tSLF. Besides, the participants in Anderson’s study were elders, while the participants in our studies were young adults. The developmental trajectories of white matter were different between bilinguals and monolinguals from early childhood to young adulthood (Pliatsikas et al., 2020). It is possible that the developmental trajectories of white matter of bilinguals and monolinguals are also different from young adulthood to old age. However, there are also some studies showed contradictory findings in the SLF. Luk et al. (2011) also recruited elder lifelong bilinguals with frequent L2 use, but they reported increased FA in the bilateral SLF. According to Anderson et al. (2018a), the matching between the groups in Luk’s study might not be enough considering the high popularity of preclinical dementia in elders. In addition, both Pliatsikas et al. (2015) and Kuhl et al. (2016) reported differences in FA of the bilateral SLF between bilinguals and monolinguals. Most of the bilinguals in the two studies started to immerse themselves in L2 environment in adulthood. The changes of FA might be related to the later exposure to L2 environment in adulthood. So we speculated that the pattern of increased MD, AD, and decreased number of streamlines in the SLF for Cantonese–Mandarin bilinguals might have relationship with the long-term frequent use of two languages from an early age.

Few previous studies on bilingualism reported the specific subdivisions of SLF. In the current study, we divided the SLF into three components and observed different structural indices in the bilateral tSLF between Cantonese–Mandarin bilinguals and Mandarin monolinguals. The tSLF connects the parietal lobe to the temporal lobe. As the function of the left and right tSLF is not the same, we will discuss them separately. The left tSLF participants in lexical retrieval and the conversion from orthography to phonology and semantics (Nakajima et al., 2019). Numerous studies reported that the proficient bilinguals showed slower lexical retrieval than comparable monolinguals (see the review of Sullivan et al., 2018). The lower number of streamlines in the left tSLF might correlate with this phenomenon. As to the right tSLF, Vaessen et al. (2016) reported the right tSLF participants in visuospatial perception. The characters of logographic languages have complex spatial structures, and character processing in logographic languages demands more involvement of brain regions related to visuospatial processing especially in the right hemisphere than alphabetic languages (Bolger et al., 2005; Tan et al., 2005). In addition, the right superior temporal gyrus that right tSLF connected is sensitive to tonal perception (Liang and Du, 2018). Loui et al. (2011) reported that FA of the right tSLF is correlated to tone learning performance. Both Cantonese and Mandarin are tonal languages. Using two tonal logographic languages might associate with white matter structural changes in the right tSLF. No significant correlations were found between the mean different DTI metrics in the right tSLF and the phonological processing skills we measured. As mentioned above, the right tSLF was related to tonal and visuospatial perception, which might explain the absence of significant correlations.

### Ventral White Matter Tracts

We also found that Cantonese–Mandarin bilinguals exhibited higher AD and FA in the left ILF, and higher AD and MD in the right IFOF. Higher FA is accompanied by more myelination, more axons, higher axonal packing density, or less fiber crossing (Basser and ÖZarslan, 2014). Higher AD was associated with more axons, increased axonal caliber, looser packing density or more coherent orientation of axons, but not to be sensitive to myelin changes (Solowij et al., 2017). The higher AD and FA in the left ILF in the bilinguals might indicate that Cantonese–Mandarin bilinguals have more axons, increased myelin or less fiber mixture. The higher AD and MD in the right IFOF might indicate increased axonal caliber or looser packing density. Consistent with our study, Luk et al. (2011) reported bilingual elders had higher FA in the bilateral ILF. Anderson et al. (2018a) reported increased AD in the left sagittal stratum including ILF and IFOF for the bilingual elders compared to the monolingual elders. Both Luk and Anderson suggested that the increased FA and AD reflected enhanced white matter integrity in bilingual elders, and this neural adaption could serve as a buffer against the neuroatrophy of aging. However, we are conservative in interpreting the change of DTI metrics as enhanced integrity, since our participants were young adults. Singh et al. (2017) observed decreased FA in the right IFOF for bilingual young adults compared to their monolingual peers. Singh et al. (2017) interpreted the FA change as lower axonal density, myelination and coherence in the orientation of white matter. The microstructure change that accompanies the decrease of FA can also induce the increase of MD; for example, lower axonal density, so our findings in the right IFOF do not contradict their results. Among these studies, the association between lifelong bilinguals’ experience and ventral white matter tracts was different because of their different age. Interesting, the participants in the current study were also young adults, but the results were consistent with that in the elder (Luk et al., 2011; Anderson et al., 2018a), so more research is needed to explore the possible reasons.

Importantly, consistent with our hypothesis, we found the differences in the ventral tracts were related to phonological processing skills. First, we found that the mean FA of the significant voxels in the left ILF was positively correlated with the IES of the Mandarin visual rhyming judgment task within the bilingual group (*r* = 0.498, *p* = 0.006), but not within the monolingual group (*F* = 6.93, *p*_interaction_ = 0.011). The correlation between the mean AD in the left ILF and IES of the Mandarin visual rhyming judgment task within bilinguals was marginally significant with *p* = 0.058 (see Supplementary Table 1). The correlations here suggested that the increased FA and AD in the left ILF might have an association with the Mandarin phonological processing for Cantonese–Mandarin bilinguals. As reported above, compared to Mandarin monolinguals, Cantonese–Mandarin bilinguals had worse performance in the Mandarin visual rhyming judgment task. We proposed two possible explanations. First, the evidence from both alphabetic and logographic languages showed that the bilateral ILF is involved in semantic processing and orthography–phonology conversion (Vandermosten et al., 2012; Herbet et al., 2018; Wang et al., 2020). To complete the visual rhyming judgment tasks, subjects need to map orthography to phonology first and then decode the phonology (McPherson et al., 1997). The left ILF might correlate to the visual rhyming task through the process of conversion from orthography to phonology. Cantonese and Mandarin use the same set of written systems, in which 70% of characters share the same orthography and meaning but not phonology in both languages, namely, cognates (Yu, 1960). Bilinguals cannot restrict the activation of phonology in the non-target language when seeing cognates, which renders language competence (Rodriguez-Fornells et al., 2005). The Cantonese–Mandarin bilinguals’ worse performance in Mandarin visual rhyming tasks might be related to language interference. The activation of semantic representations was found to facilitate the phonological accessing of cognates in L2 (Friesen et al., 2014). A previous fMRI study also reported that bilinguals who used two languages with the same orthography but different phonological associations were more likely to recruit the ventral stream than the dorsal stream in the orthography-phonology conversion in both L1 and L2 (Nosarti et al., 2010). Considering the important role of the left ILF in visual semantic processing (Shin et al., 2019), the correlation between the FA and IES may reflect the Cantonese–Mandarin bilinguals recruit the left ventral stream extra in the orthography-phonology conversion. The second possible explanation is that the correlation above may reflect the increased need for lexical retrieval for bilinguals. The Mandarin visual rhyming judgment tasks involve the process of retrieval of phonology word form. The frequency–lag hypothesis claimed that bilinguals need to divide their language use between two languages and use each language less frequently, so lexical retrieval is more effortful for bilinguals (Gollan et al., 2008, 2011). The left ILF was also reported to show a strong involvement in lexical retrieval. The correlation between FA in the left ILF and IES of the visual rhyming task may reflect the extra need for lexical retrieval for Cantonese–Mandarin bilinguals. Overall, we speculated that the increased FA and AD in the left ILF may be the result of overburden.

Second, we also found a positive correlation between the mean AD of the different voxels in the right IFOF and the IES of Cantonese auditory rhyming judgment task within the bilingual group (*r* = 0.582, *p* = 0.001). In addition, we noticed the mean AD was also correlated with IES of Mandarin auditory rhyming judgment task within the bilingual group (*r* = 0.420, *p* = 0.023), though this correlation did not survive the multiple corrections. Lebel et al. (2013) reported FA in the right IFOF/ILF was correlated with the ability of phonological decoding in healthy adults. This may explain the correlation of AD in the right IFOF with IES of the auditory rhyming tasks revealed by the current study. The Cantonese–Mandarin bilinguals use two languages frequently. Facing the competition of two languages, they may rely more on phonological decoding in auditory speech processing, which rendered a higher AD. In addition, because the IFOF connects the frontal lobe, it also plays a role in the executive function, especially in cognitive flexibility (Perez-Iglesias et al., 2010; Kucukboyaci et al., 2012) and inhibition control (Rollans and Cummine, 2018). The correlation between AD in the right IFOF and performance in auditory rhyming tasks may also reflect the bilinguals’ extra requirement of executive function. To test this hypothesis, we additionally conducted a correlation analysis between AD in the right IFOF and the measurements of executive function, which were used in our previous study (Cai et al., 2021), within the bilingual group. A positive significant correlation was found between AD and the ability of shift, namely, cognitive flexibility, within the bilingual group (*r* = –0.39, *p* = 0.04) (for the plot, see Supplementary Figure 2). The bilinguals need to switch from two languages in oral communication, and more cognitive flexibility is needed (Buchweitz and Prat, 2013). The increased recruitment of the right IFOF may bring about higher AD for the Cantonese–Mandarin bilinguals.

### Limitations

There were some limitations to this study. First, this study used a cross-sectional design which is limited to explaining the causal relationship between structural changes in white matter and long-term logographic–logographic bilingual experience. Second, the sample size was not large enough, which may affect the statistics effectiveness of the results. However, our sample size is still larger than most of the previous MRI studies on bilingualism (Luk et al., 2011; Cummine and Boliek, 2013; Gold et al., 2013; Pliatsikas et al., 2015; Kuhl et al., 2016; Singh et al., 2017). Third, English is a compulsory course in mainland China, so all participants have English experience. To minimize the confounding, we controlled their English proficiency estimated by the CET4 grades in the group-wise comparison.

## Conclusion

In conclusion, compared to Mandarin monolinguals, Cantonese–Mandarin bilinguals have different structures in the bilateral tSLF, right IFOF, and left ILF. The bilinguals’ white matter showed higher diffusivity, especially in the axonal direction, than the monolinguals. The specific difference pattern of DTI indices in the dorsal stream may reflect the neuroplasticity related to the long-term bilingual experience. As to the ventral tracts, they are not traditionally considered to participate in phonological processing. However, we found the differences in ventral white matter were related to phonological processing in Cantonese–Mandarin bilinguals. Our study confirmed the association between Cantonese–Mandarin bilingual experience and structural adaption in the ventral white matter tracts, and also the relationship between the structural adaption here and logographic language phonological processing skills in the bilinguals. Our study first provided evidence of white matter characteristics of bilinguals using two kinds of logographic languages.

## Data Availability Statement

The original contributions presented in the study are included in the article, further inquiries can be directed to the corresponding author.

## Ethics Statement

The studies involving human participants were reviewed and approved by the Medical Ethics Committee, Sun Yat-sen University. The patients/participants provided their written informed consent to participate in this study.

## Author Contributions

XX, XF, ST, and XS performed the material preparation and data collection. XX and YJ performed the data analysis. NP and MC performed the data curation. JJ performed the negotiation with the unit who helped us collect data. XL performed the supervision and the funding acquisition. XX wrote the first draft of the manuscript. All authors contributed to the study conception and design, commented on the previous versions of the manuscript, read and approved the final manuscript.

## Conflict of Interest

The authors declare that the research was conducted in the absence of any commercial or financial relationships that could be construed as a potential conflict of interest.

## Publisher’s Note

All claims expressed in this article are solely those of the authors and do not necessarily represent those of their affiliated organizations, or those of the publisher, the editors and the reviewers. Any product that may be evaluated in this article, or claim that may be made by its manufacturer, is not guaranteed or endorsed by the publisher.

## Footnotes

1
www.uottawa.ca/clmc/

2
https://fsl.fmrib.ox.ac.uk/fsl/

3
http://trackvis.org

4
quantitativeskills.com/sisa

## References

[B1] Anderson J. A. E. Grundy J. G. De Frutos J. Barker R. M. Grady C. Bialystok E. (2018a). Effects of bilingualism on white matter integrity in older adults. *Neuroimage* 167 143–150. 10.1016/j.neuroimage.2017.11.038 29175203 PMC5845836

[B2] Anderson J. A. E. Mak L. Keyvani Chahi A. Bialystok E. (2018b). The language and social background questionnaire: assessing degree of bilingualism in a diverse population. *Behav. Res. Methods* 1 250–263. 10.3758/s13428-017-0867-9 28281208 PMC5591752

[B3] Andersson J. L. R. Sotiropoulos S. N. (2016). An integrated approach to correction for off-resonance effects and subject movement in diffusion MR imaging. *Neuroimage* 125, 1063–1078. 10.1016/j.neuroimage.2015.10.019 26481672 PMC4692656

[B4] Basser P. J. Pierpaoli C. (2011). Microstructural and physiological features of tissues elucidated by quantitative-diffusion-tensor MRI. 1996. *J. Magn. Reson.* 2 560–570. 10.1016/j.jmr.2011.09.022 22152371

[B5] Basser P. J. ÖZarslan E. (2014). *Introduction to Diffusion MR.* San Diego, CA: Academic Press, 3–9.

[B6] Behrens T. E. Woolrich M. W. Jenkinson M. Johansen-Berg H. Nunes R. G. Clare S. (2003). Characterization and propagation of uncertainty in diffusion-weighted MR imaging. *Magn. Reson. Med.* 5 1077–1088. 10.1002/mrm.10609 14587019

[B7] Bolger D. J. Perfetti C. A. Schneider W. (2005). Cross-cultural effect on the brain revisited: universal structures plus writing system variation. *Hum. Brain Mapp.* 1 92–104. 10.1002/hbm.20124 15846818 PMC6871743

[B8] Bruyer R. Brysbaert M. (2011). Combining speed and accuracy in cognitive psychology: is the inverse efficiency score (IES) a better dependent variable than the mean reaction time (RT) and the percentage of errors (PE)? *Psychol. Belg.* 1 5–13. 10.5334/pb-51-1-5

[B9] Buchweitz A. Prat C. (2013). The bilingual brain: flexibility and control in the human cortex. *Phys. Life Rev.* 4 428–443. 10.1016/j.plrev.2013.07.020 23973007

[B10] Budisavljevic S. Dell’Acqua F. Zanatto D. Begliomini C. Miotto D. Motta R. (2017). Asymmetry and structure of the fronto-parietal networks underlie visuomotor processing in humans. *Cereb. Cortex* 2 1532–1544. 10.1093/cercor/bhv348 26759477

[B11] Cai L. Xu X. Fan X. Ma J. Fan M. Wang Q. (2021). Differences in brain functional networks of executive function between Cantonese-Mandarin bilinguals and Mandarin monolinguals. *Front. Hum. Neurosci.* 15:748919. 10.3389/fnhum.2021.748919 34867242 PMC8638783

[B12] Cao F. Sussman B. L. Rios V. Yan X. Wang Z. Spray G. J. (2017). Different mechanisms in learning different second languages: evidence from English speakers learning Chinese and Spanish. *Neuroimage* 148 284–295. 10.1016/j.neuroimage.2017.01.042 28110086

[B13] Cao F. Tao R. Liu L. Perfetti C. A. Booth J. R. (2013). High proficiency in a second language is characterized by greater involvement of the first language network: evidence from Chinese learners of English. *J. Cogn. Neurosci.* 10 1649–1663. 10.1162/jocn_a_00414 PMC3979436 23654223

[B14] Catani M. Allin M. P. Husain M. Pugliese L. Mesulam M. M. Murray R. M. (2007). Symmetries in human brain language pathways correlate with verbal recall. *Proc. Natl. Acad. Sci. U.S.A.* 43 17163–17168. 10.1073/pnas.0702116104 17939998 PMC2040413

[B15] Catani M. Jones D. K. Ffytche D. H. (2005). Perisylvian language networks of the human brain. *Ann. Neurol.* 1 8–16. 10.1002/ana.20319 15597383

[B16] Catani M. Thiebaut de Schotten M. (2008). A diffusion tensor imaging tractography atlas for virtual *in vivo* dissections. *Cortex* 8 1105–1132. 10.1016/j.cortex.2008.05.004 18619589

[B17] Chen X. Anderson R. C. Li W. Hao M. Wu X. Shu H. (2004). Phonological awareness of bilingual and monolingual Chinese children. *J. Educ. Psychol.* 1 142–151. 10.1037/0022-0663.96.1.142

[B18] Costa A. Sebastian-Galles N. (2014). How does the bilingual experience sculpt the brain? *Nat. Rev. Neurosci.* 5 336–345. 10.1038/nrn3709 24739788 PMC4295724

[B19] Cummine J. Boliek C. A. (2013). Understanding white matter integrity stability for bilinguals on language status and reading performance. *Brain Struct. Funct.* 2 595–601. 10.1007/s00429-012-0466-6 23097036

[B20] De Schotten M. T. Dell’Acqua F. Forkel S. Simmons A. Vergani F. Murphy D. G. (2011). A lateralized brain network for visuo-spatial attention. *Nat. Neurosci.* 14 1245–1246. 21926985 10.1038/nn.2905

[B21] Deng Y. Wu Q. Weng X. (2013). Unimodal and multimodal regions for logographic language processing in left ventral occipitotemporal cortex. *Front. Hum. Neurosci.* 7:619. 10.3389/fnhum.2013.00619 24098280 PMC3784977

[B22] Douglas R. (2008). White matter matters. *Sci. Am.* 3 54–61. 10.1038/scientificamerican0308-54 18357821

[B23] Duffau H. (2015). Stimulation mapping of white matter tracts to study brain functional connectivity. *Nat. Rev. Neurol.* 5 255–265. 10.1038/nrneurol.2015.51 25848923

[B24] Fan X. Wu Y. Cai L. Ma J. Pan N. Xu X. (2021). The differences in the whole-brain functional network between Cantonese-Mandarin bilinguals and Mandarin monolinguals. *Brain Sci.* 11:310. 10.3390/brainsci11030310 33801390 PMC8000089

[B25] Fields R. D. (2015). A new mechanism of nervous system plasticity: activity-dependent myelination. *Nat. Rev. Neurosci.* 12 756–767. 10.1038/nrn4023 26585800 PMC6310485

[B26] Friesen D. C. Jared D. Haigh C. A. (2014). Phonological processing dynamics in bilingual word naming. *Can. J. Exp. Psychol.* 3 179–193. 10.1037/cep0000026 25383476

[B27] Giacosa C. Karpati F. J. Foster N. E. V. Hyde K. L. Penhune V. B. (2019). The descending motor tracts are different in dancers and musicians. *Brain Struct. Funct.* 9 3229–3246. 10.1007/s00429-019-01963-0 31620887

[B28] Gibson E. M. Purger D. Mount C. W. Goldstein A. K. Lin G. L. Wood L. S. (2014). Neuronal activity promotes oligodendrogenesis and adaptive myelination in the mammalian brain. *Science* 6183:1252304. 10.1126/science.1252304 24727982 PMC4096908

[B29] Gold B. T. Johnson N. F. Powell D. K. (2013). Lifelong bilingualism contributes to cognitive reserve against white matter integrity declines in aging. *Neuropsychologia* 13 2841–2846. 10.1016/j.neuropsychologia.2013.09.037 24103400 PMC3856701

[B30] Gollan T. H. Montoya R. I. Cera C. Sandoval T. C. (2008). More use almost always a means a smaller frequency effect: aging, bilingualism, and the weaker links hypothesis. *J. Mem. Lang.* 3 787–814. 10.1016/j.jml.2007.07.001 19343088 PMC2409197

[B31] Gollan T. H. Slattery T. J. Goldenberg D. Van Assche E. Duyck W. Rayner K. (2011). Frequency drives lexical access in reading but not in speaking: the frequency-lag hypothesis. *J. Exp. Psychol. Gen.* 2 186–209. 10.1037/a0022256 21219080 PMC3086969

[B32] Grundy J. G. Timmer K. (2016). Bilingualism and working memory capacity: a comprehensive meta-analysis. *Second Lang. Res.* 3 325–340. 10.1177/0267658316678286

[B33] Hamalainen S. Sairanen V. Leminen A. Lehtonen M. (2017). Bilingualism modulates the white matter structure of language-related pathways. *Neuroimage* 152 249–257. 10.1016/j.neuroimage.2017.02.081 28263923

[B34] Herbet G. Zemmoura I. Duffau H. (2018). Functional anatomy of the inferior longitudinal fasciculus: from historical reports to current hypotheses. *Front. Neuroanat.* 12:77. 10.3389/fnana.2018.00077 30283306 PMC6156142

[B35] Hickok G. Poeppel D. (2007). The cortical organization of speech processing. *Nat. Rev. Neurosci.* 5 393–402. 10.1038/nrn2113 17431404

[B36] Hosoda C. Tanaka K. Nariai T. Honda M. Hanakawa T. (2013). Dynamic neural network reorganization associated with second language vocabulary acquisition: a multimodal imaging study. *J. Neurosci.* 34 13663–13672. 10.1523/jneurosci.0410-13.2013 23966688 PMC6618649

[B37] Huiming X. Zhe Z. (2016). On language use and language attitude of the youths and adolescents in Guangzhou. *Appl. Linguist.* 03 20–29.

[B38] Institute of Language, Chinese Academy of Social Sciences, Institute of Ethnology and Anthropology, Chinese Academy of Social Sciences, and Language and Information Science Research Center of City University of Hong Kong (2012). *Atlas of Chinese Language*, 2nd Edn. Beijing: The Commercial Press.

[B39] Jian H. Aitao L. Jijia Z. (2011). Cross-language interference in the verbal fluency of Mandarin-Cantanese diglossia people. *Psychol. Res.* 4 801–812.

[B40] Jin T. Kim S. G. (2008). Functional changes of apparent diffusion coefficient during visual stimulation investigated by diffusion-weighted gradient-echo fMRI. *Neuroimage* 3 801–812. 10.1016/j.neuroimage.2008.03.014 18450483 PMC2527868

[B41] Jones D. K. Knosche T. R. Turner R. (2013). White matter integrity, fiber count, and other fallacies: the do’s and don’ts of diffusion MRI. *Neuroimage* 73 239–254. 10.1016/j.neuroimage.2012.06.081 22846632

[B42] Kim S. Y. Qi T. Feng X. Ding G. Liu L. Cao F. (2016). How does language distance between L1 and L2 affect the L2 brain network? An fMRI study of Korean-Chinese-English trilinguals. *Neuroimage* 129 25–39. 10.1016/j.neuroimage.2015.11.068 26673115

[B43] Kucukboyaci N. E. Girard H. M. Hagler D. J. Jr. Kuperman J. Tecoma E. S. Iragui V. J. (2012). Role of frontotemporal fiber tract integrity in task-switching performance of healthy controls and patients with temporal lobe epilepsy. *J. Int. Neuropsychol. Soc.* 1 57–67. 10.1017/S1355617711001391 22014246 PMC3482626

[B44] Kuhl P. K. Stevenson J. Corrigan N. M. van den Bosch J. J. F. Can D. D. Richards T. (2016). Neuroimaging of the bilingual brain: structural brain correlates of listening and speaking in a second language. *Brain Lang.* 162 1–9. 10.1016/j.bandl.2016.07.004 27490686

[B45] Kuznetsova A. Brockhoff P. B. Christensen R. H. B. (2017). lmerTest package: tests in linear mixed effects models. *J. Stat. Softw.* 82 1–26. 10.18637/jss.v082.i13

[B46] Lebel C. Shaywitz B. Holahan J. Shaywitz S. Marchione K. Beaulieu C. (2013). Diffusion tensor imaging correlates of reading ability in dysfluent and non-impaired readers. *Brain Lang.* 2 215–222. 10.1016/j.bandl.2012.10.009 23290366

[B47] Li J. (1990). Cantonese is independent of Chinese. *Acad. Forum* 76, 54–76.

[B48] Li M. Zhang Y. Song L. Huang R. Ding J. Fang Y. (2017). Structural connectivity subserving verbal fluency revealed by lesion-behavior mapping in stroke patients. *Neuropsychologia* 101 85–96. 10.1016/j.neuropsychologia.2017.05.008 28495601

[B49] Li P. Legault J. Litcofsky K. A. (2014). Neuroplasticity as a function of second language learning: anatomical changes in the human brain. *Cortex* 58 301–324. 10.1016/j.cortex.2014.05.001 24996640

[B50] Li X. Yang D. Jing J. Zheng J. Luo D. Wang X. (2011). Mandarin phonological process in Mandarin-spoken university students and Cantonese-spoken university students. *Chin. Ment. Health J.* 7 528–532.

[B51] Liang B. Du Y. (2018). The functional neuroanatomy of lexical tone perception: an activation likelihood estimation meta-analysis. *Front. Neurosci.* 12:495. 10.3389/fnins.2018.00495 30087589 PMC6066585

[B52] Liu H. Cao F. (2016). L1 and L2 processing in the bilingual brain: a meta-analysis of neuroimaging studies. *Brain Lang.* 159 60–73. 10.1016/j.bandl.2016.05.013 27295606

[B53] Loui P. Li H. C. Schlaug G. (2011). White matter integrity in right hemisphere predicts pitch-related grammar learning. *Neuroimage* 2 500–507. 10.1016/j.neuroimage.2010.12.022 21168517 PMC3035724

[B54] Luk G. Bialystok E. Craik F. I. Grady C. L. (2011). Lifelong bilingualism maintains white matter integrity in older adults. *J. Neurosci.* 46 16808–16813. 10.1523/jneurosci.4563-11.2011 22090506 PMC3259110

[B55] Ma J. Fan X. Pan N. Xu X. Jin Y. Guo X. (2022). The differences of functional brain network in processing auditory phonological tasks between Cantonese-Mandarin bilinguals and Mandarin monolinguals. *Brain Res.* 1780:147801. 10.1016/j.brainres.2022.147801 35077700

[B56] Ma J. Wu Y. Sun T. Cai L. Fan X. Li X. (2020). Neural substrates of bilingual processing in a logographic writing system: an fMRI study in Chinese Cantonese-Mandarin bilinguals. *Brain Res.* 1738:146794. 10.1016/j.brainres.2020.146794 32234428

[B57] Mamiya P. C. Richards T. L. Coe B. P. Eichler E. E. Kuhl P. K. (2016). Brain white matter structure and COMT gene are linked to second-language learning in adults. *Proc. Natl. Acad. Sci. U.S.A.* 26 7249–7254. 10.1073/pnas.1606602113 27298360 PMC4932981

[B58] Mamiya P. C. Richards T. L. Kuhl P. K. (2018). Right forceps minor and anterior thalamic radiation predict executive function skills in young bilingual adults. *Front. Psychol.* 9:118. 10.3389/fpsyg.2018.00118 29479331 PMC5811666

[B59] Marian V. Shook A. (2012). *The Cognitive Benefits of Being Bilingual.* New York, NY: Dana Foundation. PMC3583091 23447799

[B60] McKenzie I. A. Ohayon D. Li H. de Faria J. P. Emery B. Tohyama K. (2014). Motor skill learning requires active central myelination. *Science* 6207 318–322. 10.1126/science.1254960 25324381 PMC6324726

[B61] McPherson W. B. Ackerman P. T. Dykman R. A. (1997). Auditory and visual rhyme judgements reveal differences and similarities between normal and disabled adolescent readers. *Dyslexia* 2 63–77. 10.1002/(SICI)1099-0909(199706)3:2<63::AID-DYS49>3.0.CO;2-Q

[B62] Nakajima R. Kinoshita M. Shinohara H. Nakada M. (2019). The superior longitudinal fascicle: reconsidering the fronto-parietal neural network based on anatomy and function. *Brain Imaging Behav.* 14 2817–2830. 10.1007/s11682-019-00187-4 31468374

[B63] Nosarti C. Mechelli A. Green D. W. Price C. J. (2010). The impact of second language learning on semantic and nonsemantic first language reading. *Cereb. Cortex* 2 315–327. 10.1093/cercor/bhp101 19478033 PMC2803733

[B64] Oldfield R. C. (1971). The assessment and analysis of handedness: the Edinburgh inventory. *Neuropsychologia* 1 97–113. 10.1016/0028-3932(71)90067-4 5146491

[B65] Parker Jones O. Green D. W. Grogan A. Pliatsikas C. Filippopolitis K. Ali N. (2012). Where, when and why brain activation differs for bilinguals and monolinguals during picture naming and reading aloud. *Cereb. Cortex* 4 892–902. 10.1093/cercor/bhr161 21705392 PMC3306575

[B66] Pennino A. M. (2010). *Monolingual and Bilingual Spanish-English Children’s Phonological Production on Rapid Automatized Naming Tasks.* Memphis, TN: University of Memphis.

[B67] Perez-Iglesias R. Tordesillas-Gutierrez D. McGuire P. K. Barker G. J. Roiz-Santianez R. Mata I. (2010). White matter integrity and cognitive impairment in first-episode psychosis. *Am. J. Psychiatry* 4 451–458. 10.1176/appi.ajp.2009.09050716 20160006

[B68] Pliatsikas C. Meteyard L. Veríssimo J. DeLuca V. Shattuck K. Ullman M. T. (2020). The effect of bilingualism on brain development from early childhood to young adulthood. *Brain Struct. Funct.* 7 2131–2152. 10.1007/s00429-020-02115-5 32691216 PMC7473972

[B69] Pliatsikas C. Moschopoulou E. Saddy J. D. (2015). The effects of bilingualism on the white matter structure of the brain. *Proc. Natl. Acad. Sci. U.S.A.* 5 1334–1337. 10.1073/pnas.1414183112 25583505 PMC4321232

[B70] Qi Z. Han M. Garel K. San Chen E. Gabrieli J. D. E. (2015). White-matter structure in the right hemisphere predicts Mandarin Chinese learning success. *J. Neurolinguist.* 33 14–28. 10.1016/j.jneuroling.2014.08.004

[B71] Rodriguez-Fornells A. van der Lugt A. Rotte M. Britti B. Heinze H. J. Munte T. F. (2005). Second language interferes with word production in fluent bilinguals: brain potential and functional imaging evidence. *J. Cogn. Neurosci.* 3 422–433. 10.1162/0898929053279559 15814002

[B72] Rollans C. Cheema K. Georgiou G. K. Cummine J. (2017). Pathways of the inferior frontal occipital fasciculus in overt speech and reading. *Neuroscience* 364 93–106. 10.1016/j.neuroscience.2017.09.011 28918257

[B73] Rollans C. Cummine J. (2018). One tract, two tract, old tract, new tract: a pilot study of the structural and functional differentiation of the inferior fronto–occipital fasciculus. *J. Neurolinguist.* 46 122–137. 10.1016/j.jneuroling.2017.12.009

[B74] Rossi E. Cheng H. Kroll J. F. Diaz M. T. Newman S. D. (2017). Changes in white-matter connectivity in late second language learners: evidence from diffusion tensor imaging. *Front. Psychol.* 8:2040. 10.3389/fpsyg.2017.02040 29209263 PMC5702476

[B75] Sagi Y. Tavor I. Hofstetter S. Tzur-Moryosef S. Blumenfeld-Katzir T. Assaf Y. (2012). Learning in the fast lane: new insights into neuroplasticity. *Neuron* 6 1195–1203. 10.1016/j.neuron.2012.01.025 22445346

[B76] Sarubbo S. De Benedictis A. Merler S. Mandonnet E. Barbareschi M. Dallabona M. (2016). Structural and functional integration between dorsal and ventral language streams as revealed by blunt dissection and direct electrical stimulation. *Hum. Brain Mapp.* 11 3858–3872. 10.1002/hbm.23281 27258125 PMC6867442

[B77] Schlaggar B. L. McCandliss B. D. (2007). Development of neural systems for reading. *Annu. Rev. Neurosci.* 30 475–503. 10.1146/annurev.neuro.28.061604.135645 17600524

[B78] Schlegel A. A. Rudelson J. J. Tse P. U. (2012). White matter structure changes as adults learn a second language. *J. Cogn. Neurosci.* 8 1664–1670. 10.1162/jocn_a_00240 22571459

[B79] Scholz J. Klein M. C. Behrens T. E. Johansen-Berg H. (2009). Training induces changes in white-matter architecture. *Nat. Neurosci.* 11 1370–1371. 10.1038/nn.2412 19820707 PMC2770457

[B80] Shin J. Rowley J. Chowdhury R. Jolicoeur P. Klein D. Grova C. (2019). Inferior longitudinal fasciculus’ role in visual processing and language comprehension: a combined MEG-DTI study. *Front. Neurosci.* 13:875. 10.3389/fnins.2019.00875 31507359 PMC6716060

[B81] Shu H. Peng H. McBride-Chang C. (2008). Phonological awareness in young Chinese children. *Dev. Sci.* 1 171–181. 10.1111/j.1467-7687.2007.00654.x 18171377

[B82] Siddaiah A. Saldanha M. Venkatesh S. K. Ramachandra N. B. Padakannaya P. (2016). Development of rapid automatized naming (RAN) in simultaneous Kannada-English biliterate children. *J. Psycholinguist. Res.* 1 177–187. 10.1007/s10936-014-9338-y 25408516

[B83] Singh N. Rajan A. Malagi A. Ramanujan K. Canini M. Della Rosa P. (2017). Microstructural anatomical differences between bilinguals and monolinguals. *Bilingualism* 21 995–1008. 10.1017/S1366728917000438

[B84] Smith S. M. (2002). Fast robust automated brain extraction. *Hum. Brain Mapp.* 3 143–155. 10.1002/hbm.10062 12391568 PMC6871816

[B85] Smith S. M. Jenkinson M. Johansen-Berg H. Rueckert D. Nichols T. E. Mackay C. E. (2006). Tract-based spatial statistics: voxelwise analysis of multi-subject diffusion data. *Neuroimage* 4 1487–1505. 10.1016/j.neuroimage.2006.02.024 16624579

[B86] Smith S. M. Nichols T. E. (2009). Threshold-free cluster enhancement: addressing problems of smoothing, threshold dependence and localisation in cluster inference. *Neuroimage* 1 83–98. 10.1016/j.neuroimage.2008.03.061 18501637

[B87] Soares J. Marques P. Alves V. Sousa N. (2013). A hitchhiker’s guide to diffusion tensor imaging. *Front. Neurosci.* 7:31. 10.3389/fnins.2013.00031 23486659 PMC3594764

[B88] Solowij N. Zalesky A. Lorenzetti V. Yücel M. (2017). “Chronic cannabis use and axonal fiber connectivity,” in *Handbook of Cannabis and Related Pathologies*, ed. Preedy V. R. (San Diego, CA: Elsevier), 391–400. 10.1016/j.pscychresns.2009.04.005

[B89] Song S. K. Sun S. W. Ramsbottom M. J. Chang C. Russell J. Cross A. H. (2002). Dysmyelination revealed through MRI as increased radial (but unchanged axial) diffusion of water. *Neuroimage* 3 1429–1436. 10.1006/nimg.2002.1267 12414282

[B90] Stein M. Winkler C. Kaiser A. Dierks T. (2014). Structural brain changes related to bilingualism: does immersion make a difference? *Front. Psychol.* 5:1116. 10.3389/fpsyg.2014.01116 25324816 PMC4183087

[B91] Su M. Zhao J. Thiebaut de Schotten M. Zhou W. Gong G. Ramus F. (2018). Alterations in white matter pathways underlying phonological and morphological processing in Chinese developmental dyslexia. *Dev. Cogn. Neurosci.* 31 11–19. 10.1016/j.dcn.2018.04.002 29727819 PMC6969203

[B92] Sullivan M. D. Poarch G. J. Bialystok E. (2018) Why is lexical retrieval slower for bilinguals? Evidence from picture naming. *Biling* 3, 479–488. 10.1017/S1366728917000694 29910667 PMC5999048

[B93] Sulpizio S. Del Maschio N. Fedeli D. Abutalebi J. (2020). Bilingual language processing: a meta-analysis of functional neuroimaging studies. *Neurosci. Biobehav. Rev.* 108 834–853. 10.1016/j.neubiorev.2019.12.014 31838193

[B94] Sun Y. Yang Y. Desroches A. S. Liu L. Peng D. (2011). The role of the ventral and dorsal pathways in reading Chinese characters and English words. *Brain Lang.* 2 80–88. 10.1016/j.bandl.2011.03.012 21546073

[B95] Takeuchi H. Taki Y. Nouchi R. Hashizume H. Sekiguchi A. Kotozaki Y. (2015). Working memory training impacts the mean diffusivity in the dopaminergic system. *Brain Struct. Funct.* 6 3101–3111. 10.1007/s00429-014-0845-2 25023736 PMC4575686

[B96] Tan L. H. Laird A. R. Li K. Fox P. T. (2005). Neuroanatomical correlates of phonological processing of Chinese characters and alphabetic words: a meta-analysis. *Hum. Brain Mapp.* 1 83–91. 10.1002/hbm.20134 15846817 PMC6871734

[B97] Tardif T. Fletcher P. Liang W. Kaciroti N. (2009). Early vocabulary development in Mandarin (Putonghua) and Cantonese. *J. Child Lang.* 5 1115–1144. 10.1017/S0305000908009185 19435545

[B98] Tsang A. Lebel C. A. Bray S. L. Goodyear B. G. Hafeez M. Sotero R. C. (2017). White matter structural connectivity is not correlated to cortical resting-state functional connectivity over the healthy adult lifespan. *Front. Aging Neurosci.* 9:144. 10.3389/fnagi.2017.00144 28572765 PMC5435815

[B99] Vaessen M. J. Saj A. Lovblad K. O. Gschwind M. Vuilleumier P. (2016). Structural white-matter connections mediating distinct behavioral components of spatial neglect in right brain-damaged patients. *Cortex* 77 54–68. 10.1016/j.cortex.2015.12.008 26922504

[B100] Vanderauwera J. Wouters J. Vandermosten M. Ghesquiere P. (2017). Early dynamics of white matter deficits in children developing dyslexia. *Dev. Cogn. Neurosci.* 27 69–77. 10.1016/j.dcn.2017.08.003 28823983 PMC6987857

[B101] Vandermosten M. Boets B. Wouters J. Ghesquiere P. (2012). A qualitative and quantitative review of diffusion tensor imaging studies in reading and dyslexia. *Neurosci. Biobehav. Rev.* 6 1532–1552. 10.1016/j.neubiorev.2012.04.002 22516793

[B102] Wagner R. K. Torgesen J. K. (1987). The nature of phonological processing and its causal role in the acquisition of reading skills. *Psychol. Bull.* 2 192–212. 10.1037/0033-2909.101.2.192

[B103] Wang K. Li X. Huang R. Ding J. Song L. Han Z. (2020). The left inferior longitudinal fasciculus supports orthographic processing: evidence from a lesion-behavior mapping analysis. *Brain Lang.* 201:104721 10.1016/j.bandl.2019.104721 31865263

[B104] Yendiki A. Koldewyn K. Kakunoori S. Kanwisher N. Fischl B. (2014). Spurious group differences due to head motion in a diffusion MRI study. *Neuroimage* 88 79–90. 10.1016/j.neuroimage.2013.11.027 24269273 PMC4029882

[B105] Yeung S. S. (2016). Cognitive mechanism underlying the relationship between rapid automatized naming and reading: a longitudinal study on Bilingua l children. *Read. Psychol.* 8 1196–1211. 10.1080/02702711.2016.1193582

[B106] Young J. M. Vandewouw M. M. Morgan B. R. Smith M. L. Sled J. G. Taylor M. J. (2018). Altered white matter development in children born very preterm. *Brain Struct. Funct.* 5 2129–2141. 10.1007/s00429-018-1614-4 29380120

[B107] Yu T. (1960). The lexicostatistic estimation of the time depths of the five main Chinese dialects. *J. Linguist. Soc. Japan* 38 33–105.

