# Supplementary material for: A Diffusion Tensor Imaging Study on the White Matter Structures Related to the Phonology in Cantonese–Mandarin Bilinguals
Source: Front Hum Neurosci. 2022 May 6;16:851669. doi: 10.3389/fnhum.2022.851669 (PMC9120590; doi:10.3389/fnhum.2022.851669)
Supplement: Supplementary file 1 [file Data_Sheet_1.docx]

**Supplemental Table1**

| **Table S1. Relationships between mean different DTI parameters of significant clusters in the TBSS analyses and phonological processing skills within either group.** | | | | | | | | | | | |
| --- | --- | --- | --- | --- | --- | --- | --- | --- | --- | --- | --- |
| Behavior measures |  | visual rhyming IES (M) | visual rhyming IES (C) | auditory rhyming IES (M) | auditory rhyming IES (C) | digit span score (M) | digit span score (C) | RAN-digit RT (M) | RAN-digit RT (C) | RAN-object RT (M) | RAN-object RT (C) |
| ***Correlations within the bilingual group*** | | | | | | | | | | | |
| ***FA*** |  |  |  |  |  |  |  |  |  |  |  |
| TBSS: ILF-L | *r* | **0.498*** | 0.349 | 0.275 | 0.265 | 0.118 | 0.04 | -0.265 | -0.225 | 0.049 | 0.196 |
|  | *p* | **0.006** | 0.064 | 0.149 | 0.165 | 0.534 | 0.833 | 0.156 | 0.242 | 0.796 | 0.307 |
| ***MD*** |  |  |  |  |  |  |  |  |  |  |  |
| Tractography: IFOF-R | *r* | -0.215 | -0.058 | -0.088 | 0.033 | -0.174 | -0.180 | 0.185 | 0.321 | 0.282 | -0.084 |
|  | *p* | 0.263 | 0.765 | 0.649 | 0.861 | 0.358 | 0.342 | 0.328 | 0.089 | 0.130 | 0.664 |
| TBSS: tSLF-R | *r* | -0.16 | -0.002 | 0.33 | 0.154 | -0.073 | -0.293 | 0.093 | 0.168 | 0.089 | -0.025 |
|  | *p* | 0.408 | 0.99 | 0.081 | 0.426 | 0.702 | 0.117 | 0.624 | 0.383 | 0.639 | 0.898 |
| ***AD*** |  |  |  |  |  |  |  |  |  |  |  |
| Tractography: IFOF-R | *r* | -0.217 | -0.210 | 0.032 | 0.023 | 0.179 | 0.043 | -0.039 | -0.040 | -0.188 | -0.419 |
|  | *p* | 0.259 | 0.275 | 0.870 | 0.907 | 0.344 | 0.823 | 0.836 | 0.838 | 0.321 | 0.024 |
| TBSS: IFOF-R | *r* | 0.24 | 0.292 | 0.42 | **0.582*** | 0.049 | -0.201 | -0.281 | -0.088 | -0.037 | 0.146 |
|  | *p* | 0.21 | 0.124 | 0.023 | **0.001** | 0.797 | 0.288 | 0.133 | 0.65 | 0.844 | 0.449 |
| TBSS: ILF-L | *r* | 0.357 | 0.304 | 0.194 | 0.385 | 0.178 | 0.003 | -0.168 | -0.04 | -0.124 | 0.174 |
|  | *p* | 0.058 | 0.109 | 0.314 | 0.039 | 0.346 | 0.989 | 0.376 | 0.836 | 0.514 | 0.366 |
| ***Number of streamlines*** | | |  |  |  |  |  |  |  |  |  |
| Tractography: tSLF-L | *r* | 0.002 | 0.082 | 0.086 | 0.170 | -0.107 | -0.173 | 0.221 | 0.162 | 0.188 | 0.114 |
|  | *p* | 0.991 | 0.674 | 0.659 | 0.379 | 0.574 | 0.362 | 0.240 | 0.402 | 0.321 | 0.557 |
| Tractography: tSLF-R | *r* | 0.022 | 0.290 | 0.315 | 0.260 | -0.132 | -0.213 | -0.035 | -0.014 | 0.095 | 0.296 |
|  | *p* | 0.910 | 0.127 | 0.096 | 0.174 | 0.486 | 0.258 | 0.854 | 0.943 | 0.618 | 0.119 |
| ***Correlations within the monolingual group*** | | | | | | | | | | | |
| Behavior measures |  | visual rhyming IES (M) | visual rhyming IES (C) | auditory rhyming IES (M) | auditory rhyming IES (C) | digit span score (M) | digit span score (C) | RAN-digit RT (M) | RAN-digit RT (C) | RAN-object RT (M) | RAN-object RT (C) |
| ***FA*** |  |  |  |  |  |  |  |  |  |  |  |
| TBSS: ILF-L | *r* | -0.187 | - | 0.139 | - | 0.064 | - | 0.05 | - | -0.118 | - |
|  | *p* | 0.351 | - | 0.488 | - | 0.735 | - | 0.792 | - | 0.536 | - |
| ***MD*** |  |  |  |  |  |  |  |  |  |  |  |
| Tractography: IFOF-R | *r* | -0.018 | - | 0.013 | - | -0.178 | - | -0.119 | - | -0.135 | - |
|  | *p* | 0.930 | - | 0.949 | - | 0.346 | - | 0.533 | - | 0.477 | - |
| TBSS: tSLF-R | *r* | 0.202 | - | -0.116 | - | 0.239 | - | 0.073 | - | -0.09 | - |
|  | *p* | 0.313 | - | 0.564 | - | 0.204 | - | 0.703 | - | 0.637 | - |
| ***AD*** |  |  |  |  |  |  |  |  |  |  |  |
| Tractography: IFOF-R | *r* | 0.180 | - | 0.201 | - | -0.234 | - | -0.287 | - | -0.390 | - |
|  | *p* | 0.370 | - | 0.314 | - | 0.214 | - | 0.124 | - | 0.033 | - |
| TBSS: IFOF-R | *r* | 0.021 | - | 0.249 | - | -0.14 | - | -0.15 | - | -0.229 | - |
|  | *p* | 0.918 | - | 0.21 | - | 0.462 | - | 0.428 | - | 0.223 | - |
| TBSS: ILF-L | *r* | -0.100 | - | -0.048 | - | 0.034 | - | -0.015 | - | 0.095 | - |
|  | *p* | 0.62 | - | 0.812 | - | 0.859 | - | 0.936 | - | 0.617 | - |
| ***Number of streamlines*** | | |  |  |  |  |  |  |  |  |  |
| Tractography: tSLF-L | *r* | -0.027 | - | -0.211 | - | -0.019 | - | -0.362 | - | 0.106 | - |
|  | *p* | 0.893 | - | 0.291 | - | 0.922 | - | 0.049 | - | 0.577 | - |
| Tractography: tSLF-R | *r* | 0.225 | - | 0.377 | - | 0.045 | - | -0.026 | - | -0.187 | - |
|  | *p* | 0.259 | - | 0.052 | - | 0.815 | - | 0.890 | - | 0.323 | - |

*Note*: The corrected *α* (the significance level) for the bilingual group was 0.010, while the corrected *α* for the monolingual group was 0.014. The significant *r* values and its correlated *p* values were in bold, while * was attached to the significant *r* values. The levels of TBSS analyses and locations of the significant clusters were shown in the column headers.

**Supplemental Table2**

| **White matter Tract** | **DTI parameter** | **Voxels** | **Peak coordinate** | ***P*_min_** |
| --- | --- | --- | --- | --- |
| tSLF (R) | MD (Bilingual>Monolingual) | 96 | (43, -48, 8) | 0.046 |
| CST (L) | MD (Bilingual>Monolingual) | 27 | (-23, -34, 43) | 0.049 |
| FMa | MD (Bilingual>Monolingual) | 19 | (2, -42, 11) | 0.049 |
| tSLF(R) | AD (Bilingual>Monolingual) | 133 | (40, -45, 19) | 0.017 |
| IFOF(R) | AD (Bilingual>Monolingual) | 369 | (40, -41, -4) | 0.014 |
| ILF(L) | AD (Bilingual>Monolingual) | 28 | (-41, -43, -7) | 0.046 |
| ILF(R) | AD (Bilingual>Monolingual) | 142 | (49, -38, -11) | 0.012 |
| ATR(L) | AD (Bilingual>Monolingual) | 28 | (-21, 16, 18) | 0.047 |
| ATR(R) | AD (Bilingual>Monolingual) | 94 | (22, 20, 8) | 0.04 |
| CST(R) | AD (Bilingual>Monolingual) | 78 | (27, -21, 13) | 0.013 |
| Hippocampal Cingulum (R) | AD (Bilingual>Monolingual) | 79 | (22, -42, -2) | 0.018 |
| FMa | AD (Bilingual>Monolingual) | 226 | (-15, -41, 13) | 0.02 |
| FMi | AD (Bilingual>Monolingual) | 91 | (14, 27, 17) | 0.039 |

*Note:* CET4 grades and average translation (mm) were controlled, with FWE corrected and a lowest threshold of 10 voxels for each cluster. Abbreviation list: temporoparietal segment of superior longitudinal fasciculus (tSLF), inferior longitudinal fasciculus (ILF), inferior fronto-occipital fasciculus (IFOF), corticospinal tract (CST), forceps minor (FMi), forceps major (FMa), anterior thalamic radiation (ATR), left (L), right (R).

**2200ms**

🔊la4

**800ms**

🔊pa4

**+**

**200ms**

**800ms**

**B**

**2200ms**

**发**

**800ms**

**它**

**+**

**200ms**

**800ms**

**A**

**Supplemental Figure 1. A represents the process of a visual rhyming judgement trial and B represents the process of an auditory rhyming judgement trial.**

In the trial of rhyming judgement task, pairs of Chinese characters were presented sequentially and subjects were instructed to judge whether the two characters or sound rhymed or not as quickly and accurately as possible. For each trial, each character and sound stimulus was presented for 800ms, with a 200ms blank interval before the second character. A red fixation cross appeared on the screen immediately after the offset of the second character in the stimuli pair, indicating the need to make a response. The response interval duration was set at 2200ms, such that each trial lasted for 4000ms. Subjects were required to make a “yes” response with their right index finger or “no” response with their left index finger immediately when the red fixation cross appeared. For the auditory materials, we had two different speakers for the two languages. A native Mandarin speaker and a native Cantonese speaker who majored in broadcasting recorded the Mandarin and Cantonese listening materials, respectively.


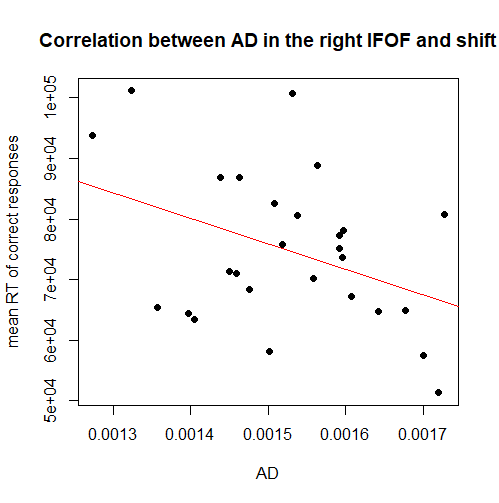


**Supplemental Figure 2. Correlation between the mean AD of the different voxels in the right IFOF and the shift score (*r*=-0.39, *P*=0.04). The shift task was used in our previous study (Cai, et al., 2021). The shift score was calculated as the mean reaction time of correct responses.**
